# The Complete Mitochondrial Genomes of Four Species in the Subfamily Limenitidinae (Lepidoptera, Nymphalidae) and a Phylogenetic Analysis

**DOI:** 10.3390/insects13010016

**Published:** 2021-12-22

**Authors:** Ning Liu, Lijun Fang, Yalin Zhang

**Affiliations:** 1 Key Laboratory of Plant Protection Resources and Pest Management, Ministry of Education, Entomological Museum, College of Plant Protection, Northwest A&F University, Xianyang 712100, China; ln-liuning@nwafu.edu.cn; 2 Shaanxi Engineering Research Centre for Conservation and Utilization of Botanical Resources, Xi’an Botanical Garden of Shaanxi Province (Institute of Botany of Shaanxi Province), Xi’an 710061, China; fanglijunn@hotmail.com

**Keywords:** Limenitidinae, butterfly, mitogenome, phylogeny

## Abstract

**Simple Summary:**

As it is currently delineated, the subfamily Limenitidinae (Lepidoptera: Nymphalidae) is comprised of 50 genera with approximately 1100 species. The classification of this subfamily has always been unstable. There are tribes and genera whose status is doubtful. Their phylogenetic relationships are far from being clarified and the monophyly of some of them is under question. To provide further insight into the relationships among included tribes, four newly-completed mitochondrial genomes of Limenitidinae (*Neptis thisbe*, *Athyma zeroca*, and *Aldania raddei*) have been sequenced and analyzed. Results indicate that the gene orientation and arrangement are similar to typical mitogenomes in Lepidoptera. The inferred phylogenetic analysis shows that tribe levels are well-supported monophyletic groups. Taken together, this work will provide a well-resolved framework for future study of this subfamily.

**Abstract:**

The complete mitogenomes of four species, *Neptis thisbe*, *Neptis obscurior*, *Athyma zeroca*, and *Aldania raddei*, were sequenced with sizes ranging from 15,172 bp (*N. obscurior*) to 16,348 bp (*Al. raddei*). All four mitogenomes display similar nucleotide content and codon usage of protein-coding genes (PCGs). Typical cloverleaf secondary structures are identified in 21 tRNA genes, while *trnS1* (AGN) lacks the dihydrouridine (DHC) arm. The gene orientation and arrangement of the four mitogenomes are similar to that of other typical mitogenomes of Lepidoptera. The Ka/Ks ratio of 13 PCGs among 58 Limenitidinae species reveals that *cox1* had the slowest evolutionary rate, while *atp8* and *nad6* exhibited a higher evolutionary rate. The phylogenetic analysis reveals that tribe-levels are well-supported monophyletic groups. Additionally, Maximum Likelihood analysis recovered the relationship (Parthenini + ((Chalingini + (Cymothoini + Neptini)) + (Adoliadini + Limenitidini))). However, a Bayesian analysis based on the same dataset recovered the relationship (Parthenini + (Adoliadini + ((Cymothoini + Neptini) + (Chalingini + Limenitidini)))). These results will offer valuable data for the future study of the phylogenetic relationships for Limenitidinae.

## 1. Introduction

The subfamily Limenitidinae (Lepidoptera: Nymphalidae) is comprised of 1100 described extant species belonging to 50 genera (http://www.nymphalidae.net/Nymphalidae/General/Limenitidinae.htm, accessed on 6 June 2021). Limenitidinae are widely distributed in all major biogeographical regions including the Nearctic, Neotropical, Palaearctic, Afrotropical, Oriental and Australasian realms [1,2,3]. Classification at the level of this subfamily, as well as lower levels within this subfamily has always been unstable [4]. Based on phenotypic traits, the Limenitidinae of the world are classified into one to three tribes [4]. Based on morphological characters, Asian Limenitidinae are divided into five tribes including Parthenini, Euthaliini, Neptini, Chalingini and Limenitini [2]. Recent phylogenetic analysis based on molecular datasets supports seven tribes within Limenitidinae, including the four traditional tribes (Parthenini, Neptini, Limenitini and Adoliadini) and three new tribes (Cymothoini, Pseudoneptini and Pseudacraeini) [4]. The previous phylogenetic relationships within Limenitidinae mainly reflected the morphological characters or/and several gene fragments. Whole mitogenomes have been used widely for inferring population genetics, phylogeography, and molecular systematics at different taxonomic scales [5]. In recent years, mitochondrial genomes can be quickly and economically obtained by using next-generation sequencing (NGS) approaches. A study on the relationships among four tribes (Parthenini, Neptini, Adoliadini, and Limenitidini) of Limenitidinae using mitochondrial genomes revealed that *Athyma* was not a monophyletic group [6]. Subsequently, the same method was used to infer relationships of genera in Limenitidini [7].

In this study, we sequenced and annotated the complete mitochondrial genomes of four species belonging to Limenitidinae including *Neptis thisbe* (GenBank accession no. OK393687), *Neptis obscurior* (GenBank accession no. OK393686), *Athyma zeroca* (GenBank accession no. OK393685) and *Aldania raddei* (GenBank accession no. OK393684). A total of 61 mitochondrial genomes (four new mitogenomes, 54 previously published mitochondrial genomes of Limenitidinae and three outgroups of Heliconiinae) were used to reconstruct phylogenetic trees based on the concatenated nucleotide sequences of several different datasets to explore the impacts of inclusion or exclusion of RNA genes on the phylogenetic resolution. The mitochondrial structure of these four species was also analyzed. The purpose of this study was to test the monophyly of tribes and analyze phylogenetic relationships among major lineages of this subfamily.

## 2. Materials and Methods

### 2.1. Sample Collection, Identification and DNA Extraction

The adults of *N. thisbe*, *N. obscurior*, *At. zeroca*, and *Al. raddei* for genome sequencing were collected in China and Table 1 includes the detailed information. Legs or thoracic muscle tissue taken from fresh specimens were immediately placed in absolute ethanol and then preserved at −20 °C for further sequencing. Collected specimens were identified based on morphological descriptions and illustrations (especially the male genitalia) [2]. Voucher specimens were deposited in the Entomological Museum of the Northwest A&F University, Yangling, Shaanxi Province, China. EasyPure R Genomic DNA Kit (TransGen Biotech, Beijing, China) was used to isolate total DNA from legs or thoracic muscle tissue following the manufacturer’s protocols. The DNA was stored at −20 °C for further analysis.

### 2.2. Mitogenome Sequencing and Assembly

Libraries were prepared by steps of shearing the total DNA by ultrasound (Covaris), end-repair, A-tailing, ligating index adapters and PCR amplification. The sequencing was carried out on the Illumina Hiseq^TM^ Xten platform (Novogene Technologies, Beijing, China) with the strategy of 150 bp paired-ends. Quality control was implemented using the software FastQC (http://www.bioinformatics.babraham.ac.uk/projects/fastqc, accessed on 8 May 2021). Geneious 8.1.3 (Biomatters, Auckland, New Zealand) was used to reconstruct mitogenomes using the clean paired reads with default parameters and the mitogenome of *Neptis philyra* (GeneBank accession number: NC_024419) as the reference.

### 2.3. Mitogenome Annotation and Sequence Analyses

Geneious v8.1.3 was used to annotate the mitogenomes with default parameters. Protein-coding genes (PCGs) and rRNA genes were annotated by alignment with the homologous sequence from *Neptis philyra* (GeneBank accession number: NC_024419) based on the invertebrate mitochondrial genetic code. The tRNA genes were identified by the MITOS server (http://mitos.bioinf.uni-leipzig.de/index.py, accessed on 10 July 2021) [8]. Secondary structures for tRNAs were manually drawn with Adobe Illustrator 2021 according to the MITOS predictions. The circular mitogenomic maps were visualized using the CGView server (http://stothard.afns.ualberta.ca/cgview_server/, accessed on 19 July 2021) [9]. PhyloSuite v1.2.2 [10] was used to calculate the nucleotide composition and skew, codon usage of PCGs and relative synonymous codon usage (RSCU) values of each PCG. Tandem Repeats Finder (http://tandem.bu.edu/trf/trf.html, accessed on 16 August 2021) [11] was applied to predict tandem repeat units of the A + T-control region. Strand asymmetry was calculated by the formulas: AT-skew = [A − T]/[A + T] and GC-skew = [G − C]/[G + C]. A sliding window analysis (a sliding window of 200 bp and step size of 20 bp) was performed to determine nucleotide diversity (Pi value) of PCGs among Limenitidinae mitogenomes using DnaSP v6. DnaSP v6 was also applied to determine the rate of non-synonymous (Ka) and synonymous (Ks) substitution rates for each PCG. Under the Kimura 2-parameter model, MEGA X [12] was used to calculate the average genetic distance of each PCG among 58 Limenitidinae species. The genetic distances and Ka/Ks ratios were graphically plotted using GraphPad Prism v8.0.1. All newly-sequenced mitogenomes were submitted to GenBank with the following accession numbers: OK393684–OK393687 (Table S1).

### 2.4. Phylogenetic Analyses

A dataset of 61 mitogenomes of Nymphalidae was used in the phylogenetic analyses. Four newly-sequenced mitogenome sequences and another 54 published available mitogenome sequences of Limenitidinae in the NCBI database were selected as ingroups (Table S1), while three species from Heliconiinae were selected as outgroups (Table S1).

PhyloSuite v1.2.2 was used to extract PCGs and RNAs. MAFFT v7.313 integrated into PhyloSuite v1.2.2 was used to align each PCG in batches with codon alignment mode and G-INS-I (accurate) strategy. All RNAs were aligned using the MAFFT 7 online service (https://mafft.cbrc.jp/alignment/server/, accessed on 18 May 2021) with the Q-INS-I algorithm [13]. Gblocks 0.91b [14] was used to remove poorly aligned regions. Substitution saturation of each dataset was analyzed using the index of substitution saturation (Iss) of Xia [15] in DAMBE 7 [16]. All aligned genes were concatenated using PhyloSuite v1.2.2. 

In order to evaluate the effect of data partitioning and incorporation of RNAs on phylogeny, three different datasets were generated, including the PCG123 dataset (13 PCGs), the PCG123R dataset (13 PCGs and two rRNAs) and the PCG123RT dataset (13 PCGs, two rRNAs and 22 tRNAs). The generated datasets were further partitioned by using PartitionFinder 2.1.1 (www.phylo.org, accessed on 2 June 2021) [17] with the “greedy” search algorithm and Bayesian Information Criterion (BIC). Details of the best-fit schemes calculated for each dataset are shown in Table S4. Topologies on the datasets were compared using the phylogenetic methods of Maximum Likelihood (ML) and Bayesian Inference (BI). IQ-TREE v.1.6.8 [18] was used to perform ML analysis under an edge-linked partition model. Bootstrap support (BS) was assessed using 5000 ultrafast bootstrap (UFB) replicates [19]. BI analysis was implemented in the CIPRES Science Gateway (www.phylo.org, accessed on 18 July 2021) with MrBayes 3.2.6 (www.phylo.org, accessed on 18 July 2021) [20]. The analyses of each dataset were performed with four chains and run for 20 million generations. Every 1000 generation was sampled as a consensus tree. The convergence of the independent runs was indicated by a standard deviation of split frequencies <0.01 and an estimated sample size (ESS) > 200. When two independent runs were mixed well, the first 25% of sampled trees were discarded following the default settings and the remaining trees were used to represent the values of posterior probability (PP).

## 3. Results and Discussion

### 3.1. General Features, Gene Order and Base Composition

**Figure 1:**
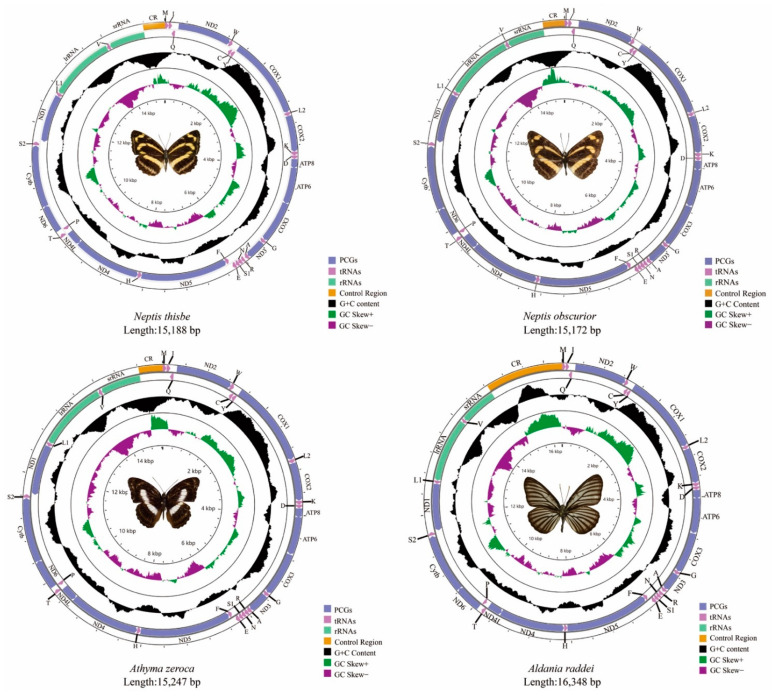
The mitogenomes of Neptis thisbe, Neptis obscurior, Athyma zeroca, and Aldania raddei.

The total size of the mitogenomes of *N. thisbe*, *N. obscurior*, *At. zeroca*, and *Al. raddei* are 15,188 bp, 15,172 bp, 15,247 bp and 16,348 bp, respectively (Figure 1). *Al. raddei* had the longest sequence length, whereas *N. obscurior* had the shortest. Length differences of mitogenomes were mainly due to the variable size of the A + T-control region. The length of these new and other published mitogenomes is quite conserved at between 15–16 kb.

Each newly-sequenced mitogenome is composed of 37 genes (13 PCGs, two rRNAs and 22 tRNAs) and a non-coding A + T-control region. Among the 37 genes in these four mitogenomes, 23 genes (nine PCGs and 14 tRNAs) were located on the majority strand (J-strand) while the minority strand (N-strand) encoded another 14 genes (four PCGs, two rRNAs and eight tRNAs) (Table S2). The gene order and orientation of these four mitogenomes were identical to typical mitogenomes of Lepidoptera [21,22]. Compared to the common type that has been found in insects [23,24], the movement of tRNA^Met^ to a position 5*′*-upstream of tRNA^Ile^ in lepidopteran insects results in the order of tRNA^Met^, tRNA^Ile^, and tRNA^Gln^ [22].

The base composition of *N. thisbe* was A = 38.0%, C = 12.6%, G = 8.2% and T = 41.1%; *N. obscurior* was A = 38.3%, C = 12.5%, G = 8.0% and T = 41.2%; *At. zeroca* was A = 38.4%, C = 11.5%, G = 7.8% and T = 42.3% and A = 38.6%, C = 12.1%, G = 8.2% and T = 41.2% in *Al. raddei*. The mitogenomes of the four species possesses a significant AT bias with the nucleotide composition ranging from 79.1% (*N. thisbe*) to 80.7% (*At. zeroca*) (Table S3), which is typical for Lepidoptera [25,26]. The AT-skew ranges from −0.048 (*At. zeroca*) to −0.033 (*Al. raddei*) and the GC-skew ranges from −0.220 (*N. obscurior*) to −0.192 (*At. zeroca*).

The PCGs have the lowest AT content. However, the control region has the highest AT content except for *Al. raddei* (80%). Besides, the AT content in rRNAs is higher than PCGs and tRNAs in these four species (Table S3).

### 3.2. Protein-Coding Genes

The total size of the 13 PCGs of *N. thisbe*, *N. obscurior*, *At. zeroca* and *Al. raddei* are 11,173 bp, 11,179 bp, 11,203 bp and 11,188 bp, respectively (Table S3). Of the 13 PCGs, nine are located on the J-strand while the other four PCGs are encoded by the N-strand. The AT-skew ranges from −0.0391 (*At. zeroca*) to −0.0141 (*N. obscurior*) and the GC-skew ranges from −0.2127 (*N. obscurior*) to −0.1787 (*At. zeroca*) in these four species. Except for *cox1* that begins with CGA, all other PCGs initiated strictly with ATN as the start codon (ATA, ATT and ATG) (Table S2). The CGA as the start codon of the *cox1* is a common phenomenon in Lepidoptera mitogenomes [27,28,29,30,31,32,33]. Most of the PCGs terminate with a TAA or TAG, while *cox1*, *cox2*, *nad4* and *nad5* terminate with an incomplete T residue. These incomplete termination codons are presumed to be filled by polyadenylation during the mRNA maturation process [34].

**Figure 2:**
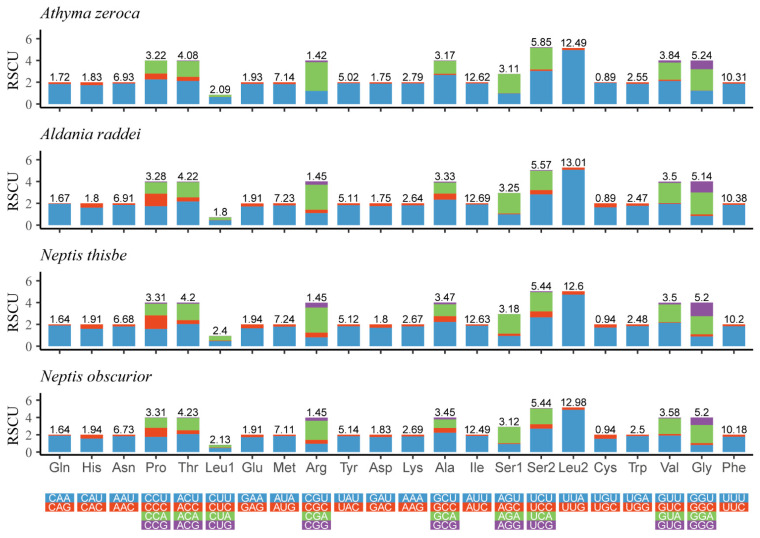
Relative synonymous codon usage (RSCU) of the mitogenomes of four Limenitidinae species. The ordinate represents the RSCU (the number of times a certain synonymous codon is used/the average number of times that all codons encoding the amino acid are used). The abscissa represents different amino acids. The number above the bar graph represents the ratio of amino acids (number of certain amino acids/total number of all amino acids). Termination codons were excluded in the study.

The Relative Synonymous Codon Usage (RSCU) of four newly-determined mitogenomes is shown in Figure 2. The five most frequently used codons are UUU (Phe), UUA (Leu), AUU (Ile), AUA (Met), and AAU (Asn). All of these observations indicate a strong AT bias of the protein-coding genes in these four mitogenomes.

### 3.3. Transfer RNA, Ribosomal RNA Genes and Non-Coding Regions

As expected, each mitogenome of the four species contained 22 typical tRNAs. The 14 tRNAs were encoded by the J-strand and the remaining eight were located on the N-strand. Their length in the four newly-sequenced mitogenomes ranged from 63 bp (*trnC*, *trnR*, *trnS1*) to 71 bp (*trnK*, *trnH*) in *N. thisbe*, from 61 bp (*trnS1*) to 71 bp (*trnK*, t*rnH*) in *N. obscurior*, from 61 bp (*trnS1*) to 71 bp (*trnK*) in *At. zeroca* and from 61 bp (*trnS1*) to 71 bp (*trnK*, *trnH*) in *Al. raddei* (Table S2).

Except for *trnS1*, all the tRNA genes showed a canonical cloverleaf secondary structure. The secondary structure of *trnS1* lacked the dihydrouridine (DHU) arm (Figure S1), and the lack of the DHU arm in *trnS1* is common in metazoan mitogenomes [5,35]. The amino acid acceptor and anticodon arms are highly conserved, while the DHU and pseudouridine (TΨC) arms are variable. A total of six types of unmatched base pairs (GU, UU, CU, AC, and single A and U) of tRNAs were found in the four new mitogenomes.

The *16S* rRNAs (*rrnL*) of all four mitogenomes were located at the intergenic region between *trnL1* and *trnV* with the length varying from 1314 bp to 1329 bp. The *12S* rRNAs (*rrnS*) were located between *trnV* and the A + T rich region with the size ranging from 723 bp to 775 bp. The two rRNA genes (*rrnL* and *rrnS*) were encoded on the N-strand in four mitogenomes. These two rRNAs have a high AT bias that reaches 84.1% in *N. thisbe*, 84.1% in *N. obscurior*, 84.9% in *At. zeroca* and 80.5% in *Al. raddei*.

The A + T-rich region is also called the control region (CR) located between *rrnS* and *trnM*. The full lengths of the CR were 407 bp in *N. thisbe*, 397 bp in *N. obscurior*, 429 bp in *At. zeroca* and 1501 bp in *Al. raddei* with the AT content ranging from 80% (*Al. raddei*) to 95.2% (*N. obscurior*) (Table S3). As in other lepidopteran mitogenomes [27,36], the A + T-rich region of each mitogenome contains the motif ATAGA which is located between the 5′-end of the *rrnS* and poly-T stretch and is the origin of the minority strand replication.

### 3.4. Gene Overlaps and Intergenic Spacers

Gene overlaps are present in all four mitogenomes and each single overlap ranges from 1 bp to 8 bp (*N. thisbe*, 11 gene junctions, 30 bp overlaps; *N. obscurior*, 12 gene junctions, 35 bp overlaps; *At. zeroca*, 12 gene junctions, 31 bp overlaps; *Al. raddei*, 10 gene junctions, 31 bp overlaps, respectively). All four species share the same 10 gene overlaps: *trnI*-*trnQ* (3 bp), *nad2*-t*rnW* (2 bp), *trnW*-*trnC* (8 bp), *trnK*-*trnD* (1 bp), *atp8*-*atp6* (7 bp), *nad3*-*trnA* (2 bp), *trnN*-*trnS1* (2 bp), *trnE*-*trnF* (2 bp), *nad4*-*nad4L* (1 bp) and *nad6*-*cytb* (1 bp).

Intergenic spacers were identified in the four mitogenomes including 11 intergenic spacers in *N. thisbe*, 13 in *N. obscurior*, 12 in *At. zeroca* and 11 in *Al. raddei*. The size of these intergenic spacers ranges from 1 bp to 57 bp and the longest intergenic spacer is located between *trnQ* and *nad2* (Table S2). The intergenic spacer found in most lepidopteran mitogenomes [27] seems to be fundamental to the recognition of the transcription termination site by the transcriptional machinery [27,37].

### 3.5. Nucleotide Diversity and Evolutionary Rate Analysis

**Figure 3:**
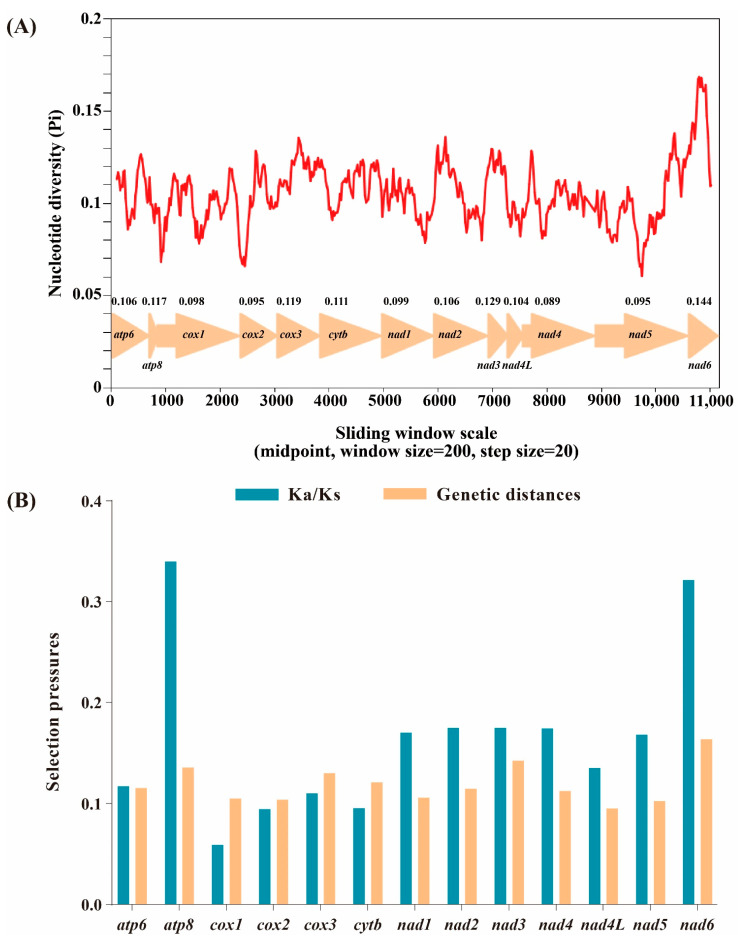
Nucleotide diversity and selection pressures on 13 PCGs in Limenitidinae. (**A**) Sliding window analysis of the alignment of 13 protein-coding genes. The value of nucleotide diversity (Pi) is indicated by the red curve. Pi values and genes are indicated below the red curve. (**B**) Genetic distances and non-synonymous (Ka) to synonymous (Ks) substitution rates of 13 protein-coding genes among 58 Limenitidinae species.

Based on 13 aligned PCGs of 58 Limenitidinae species, the nucleotide diversity was calculated (Figure 3A). The *nad6* gene is the most variable region with the highest nucleotide diversity (Pi = 0.144), while the *nad4* gene is the most conserved with the lowest value (Pi = 0.089).

The evolutionary rate was estimated by the ratio of Ka/Ks (ω) of 13 PCGs of 58 Limenitidinae mitogenomes (Figure 3B). The ratio of Ka/Ks less than, equal to and greater than 1 shows that genes are under negative (purifying) selection, neutral evolution and positive (adaptative) selection, respectively [38]. All 13 PCGs display low evolutionary rates (0 < ω < 1), suggesting that these genes experienced purifying selection. *Cox1* has undergone the strongest purifying selection and exhibits the lowest evolutionary rate (ω = 0.009). By contrast, *atp8* (ω = 0.726) and *nad6* (ω = 0.442) are likely to be under a relaxed purifying selection, indicating a relatively fast evolutionary rate.

Mitochondria play a critical role in energy production. Non-synonymous substitutions are generally harmful by reducing the efficiency of metabolic processes [39]. Highly effective purifying selection is triggered by the harmful effect of mitochondrial non-synonymous mutation to maintain the fitness of the mitogenome [40]. As flying species, butterflies rely on efficient energy supply, which may be the reason why 13 PCGs of 58 Limenitidinae mitogenomes experienced purifying selection to maintain function.

### 3.6. Phylogenetic Relationships

**Figure 4:**
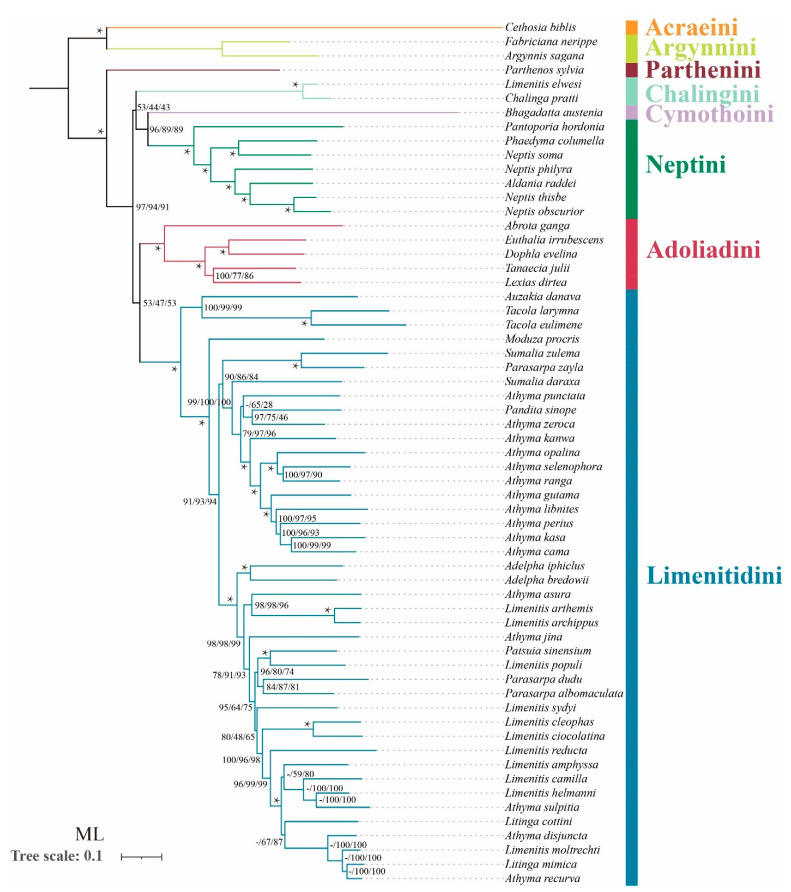
Phylogenetic tree produced by maximum likelihood analyses based on the datasets of PCG123, PCG123R and PCG123RT. Supports at nodes (from left to right) are bootstrap support values (BS) for PCG123, PCG123R and PCG123RT. “-” indicates the clades are different. Star symbol indicates that three datasets produced a maximum support value.

**Figure 5:**
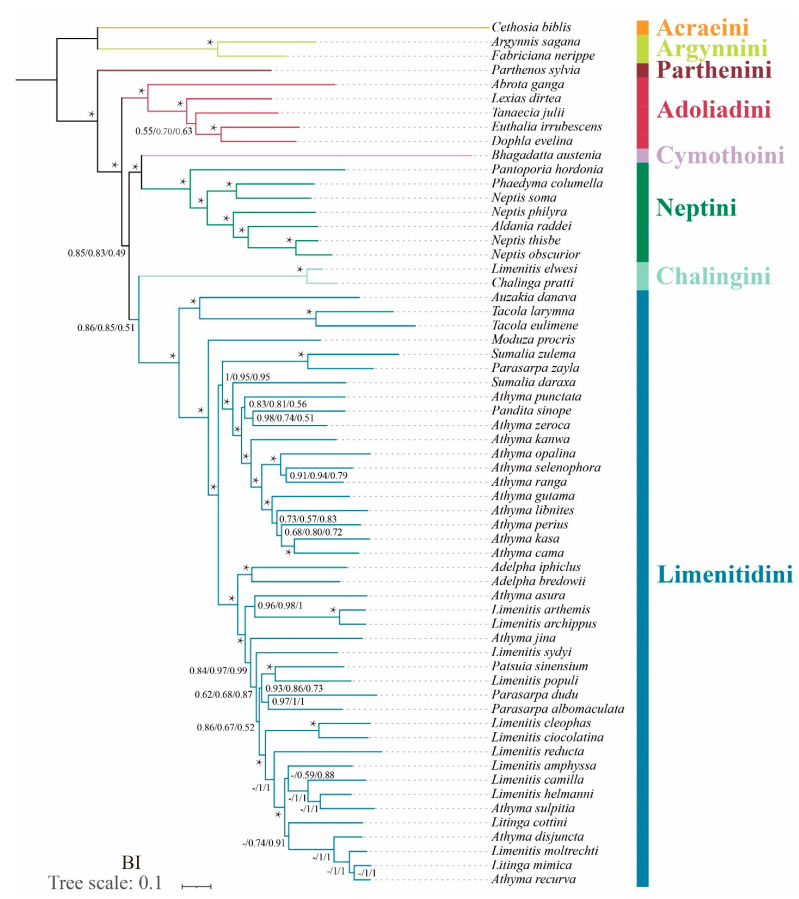
Phylogenetic tree produced by Bayesian inference analyses based on the datasets of PCG123, PCG123R and PCG123RT. Supports at nodes (from left to right) are posterior probability (BPP) for PCG123, PCG123R and PCG123RT. “-” indicates the clades are different. Star symbol indicates that three datasets produced a maximum support value.

In terms of tribal-level relationships, three datasets (PCG123, PCG123R and PCG123RT) yield identical topologies based on the same phylogenetic methods. The subfamily Limenitidinae as a monophyletic clade is recovered by both the ML (Figure 4) and BI (Figure 5) methods with strong bootstrap support (BS = 100) and high posterior probabilities (PP = 1.0). The relationships of tribes within the subfamily Limenitidinae are all monophyletic and well-supported. In BI analyses, the Parthenini + (Adoliadini + ((Cymothoini + Neptini) + (Chalingini + Limenitidini))) is recovered. In ML analyses, their relationships are Parthenini + ((Chalingini + (Cymothoini + Neptini)) + (Adoliadini + Limenitidini)). Topology discrepancies tend to occur in branches with low support, which may be responsible for the differences between BI and ML analyses. Increasing additional mitogenome sampling may be helpful to solve this problem in the future.

Parthenini as a sister to the rest of Limenitidinae was recovered by both the ML and BI methods with strong support. The results of the phylogenetic relationships are in accordance with previous publications [3,6,41,42,43].

The monophyly of Adoliadini is strongly supported in six trees (BS = 100, PP = 1.0). The position of Adoliadini as sister to the ((Cymothoini + Neptini) + (Chalingini + Limenitidini)) is stable across BI. However, the Adoliadini is placed as a sister group to Limenitidini in ML analyses with lower support, which is consistent with previous studies based on mitogenome and morphological characters [6,43].

Remarkably, *Bhagadatta austenia* was placed in the tribe Limenitidini by Harvey [44] and Wu et al. [6]. Willmott noted similarities in genitalia between *Bhagadatta austenia* and *Cymothoe*, and therefore, *Bhagadatta austenia* was placed incertae sedis [3]. However, some scholars have moved it into Cymothoini on the basis of *cox1* and multigene dataset [4,45]. In this current study, *Bhagadatta austenia* was classified in the tribe Cymothoini which is placed as a sister group to Neptini with high values (BS = 96, PP = 1.0) (Figure 4 and Figure 5).

Neptini forms a strongly supported (BS = 100, PP = 1.0) monophyletic group with *Pantoporia* being sister to the rest of Neptini. However, Neptis is not a monophyletic group with *Phaedyma columella* and *Aldania raddei* being within *Neptis*. These results are consistent with previous studies [4,42,46,47,48].

The *Chalinga pratti* (also known as *Seokia pratti*) and *Limenitis elwesi* (also known as *Chalinga elwesi*) are classified into the Chalingini [2,49] based on the morphological features of veins. Willmott suspected that *Chalinga* (including *Seokia*) perhaps did not belong to Limenitidinae [3]. In this study, the clade of *Chalinga pratti* and *Limenitis elwesi* is positioned as sister group to the core Limenitidini with low support (PCG123, PP = 0.86; PCG123R, PP = 0.85; PCG123RT, PP = 0.51, respectively) in all the BI trees, which is in line with a previous study [4]. However, the position of *Chalinga pratti* and *Limenitis elwesi* as sister to the Cymothoini and Neptini is stable across ML analyses, it also has low support values (PCG123, BS = 53; PCG123R, BS = 44; PCG123RT, BS = 43 respectively). It is worth mentioning that *Chalinga pratti* is sister to Limenitis elwesi with strong support values (BS = 100, PP = 1.0) in all analyses.

Though mitogenomes are widely used to infer phylogenetic relationships between Lepidoptera taxa. However, there are limitations to only relying on a single mitochondrial genome data. *Wolbachia* is an intracellular bacterium infecting many insect species and spreading by diverse horizontal and vertical means. As co-inherited organisms, these bacteria often lead to divergences in mitochondrial phylogenies, such as in butterflies [50,51]. Large integrated datasets are required to improve phylogenetic resolution between Lepidoptera taxa in the future, such as greater integrated datasets of nuclear genes, mitogenomes and morphological characters.

## 4. Conclusions

In this study, four newly complete mitogenomes of *N. thisbe*, *N. obscurior*, *At. zeroca*, and *Al. raddei* were sequenced and analyzed. We found that the gene orientation and arrangement of the four mitogenomes are similar to that of other typical mitogenomes of Lepidoptera. The *nad6* and *atp8* could be selected as potential DNA markers for species delimitation and clarifying phylogenetic relationships among Limenitidinae species. Phylogenetic analyses based on concatenating three datasets (PCG123, PCG123R and PCG123RT) will provide a well-resolved framework of phylogeny for Limenitidinae. In future research, it is necessary to increase the taxon sampling to test the monophyly of these genera.

## Figures and Tables

**Table 1:** The voucher information of the specimens used for mitochondrial genomes sequencing in this study.

Species	Location	Collection Date
*Neptis thisbe*	Qinling Mountain, Shaanxi	2 July 2020
*Neptis obscurior*	Qinling Mountain, Shaanxi	15 June 2020
*Athyma zeroca*	Shuanglonggou, Guangxi	12 July 2019
*Aldania raddei*	Qinling Mountain, Shaanxi	26 May 2020

## Data Availability

The data presented in this study are available on request from the corresponding author.

## Supplementary Materials

The following are available online at https://www.mdpi.com/article/10.3390/insects13010016/s1, Table S1: List of taxa used for the phylogenetic analyses in this study, Table S2: Mitogenomic organization of *Neptis thisbe*, *Neptis obscurior*, *Athyma zeroca* and *Aldania raddei*, Table S3: Nucleotide compositions in regions of the *Neptis thisbe*, *Neptis obscurior*, *Athyma zeroca* and *Aldania raddei* mitochondrial genomes, Table S4: Best partitioning scheme and nucleotide substitution models for different datasets selected by PartitionFinder. Figure S1: Predicted secondary cloverleaf structure for the tRNAs of *Neptis thisbe*, *Neptis obscurior*, *Athyma zeroca*, and *Aldania raddei*.
