# Supplementary material for: The Complete Mitochondrial Genomes of Four Species in the Subfamily Limenitidinae (Lepidoptera, Nymphalidae) and a Phylogenetic Analysis
Source: Insects. 2021 Dec 22;13(1):16. doi: 10.3390/insects13010016 (PMC8781921; doi:10.3390/insects13010016)
Supplement: Supplementary file 1 [file insects-13-00016-s001.zip › insects-1513047-supplementary.pdf]

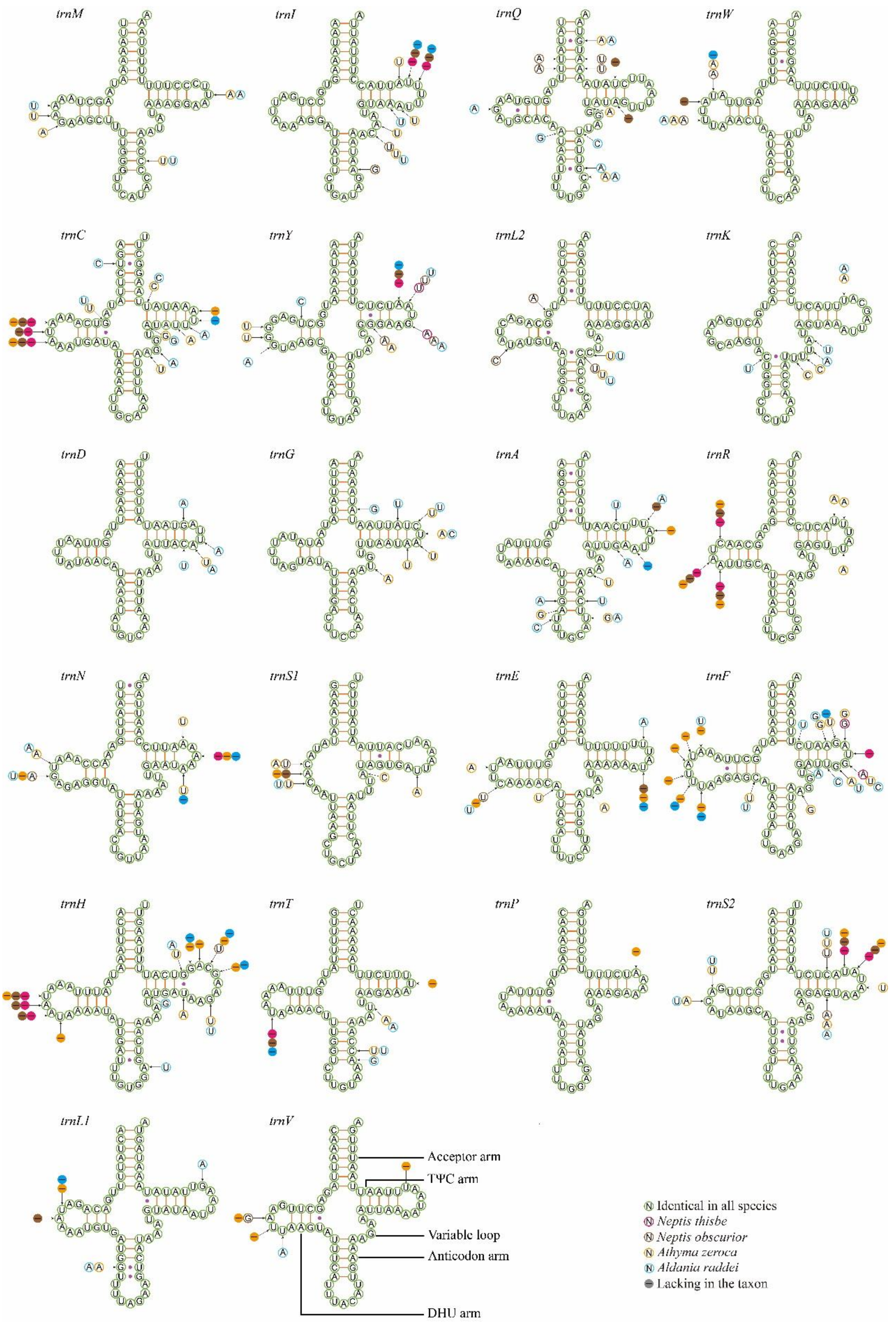

Figure S1. Predicted secondary cloverleaf structure for the tRNAs of *Neptis thisbe*, *Neptis obscurior*, *Athyma zeroca,* and *Aldania raddei*.

Table S1. List of taxa used for the phylogenetic analyses in this study.

Species	GenBank no.	Size (bp)	Total A+T%	References
Nymphalidae: Heliconiinae				
Cethosia biblis	NC_026070	15,211	79.8	Unpublished
Damora sagana	NC_037006	15,151	81	[33]
Fabriciana nerippe	NC_016419	15,140	80.9	[22]
Nymphalidae: Limenitidinae				
Athyma sulpitia	NC_017744	15,268	81.9	[30]
Athyma asura	NC_024410	15,181	81.1	[6]
Athyma cama	NC_024395	15,269	80.6	[6]
Athyma kasa	NC_024394	15,230	80.3	[6]
Athyma opalina	NC_024418	15,240	81.1	[6]
Athyma perius	NC_024397	15,277	79.6	[6]
Athyma selenophora	NC_024393	15,200	81.3	[6]
Athyma ranga	NC_039883	15,265	80.8	[6]
Athyma punctata	NC_039872	15,240	80.1	[7]
Athyma recurva	NC_039873	15,189	81	[7]
Athyma jina	NC_039699	15,202	80.8	[7]
Athyma kanwa	NC_039697	15,278	80.9	-
Athyma disjuncta	NC_039876	15,198	81.2	[7]
Tarattia libnites	NC_039886	15,216	80.8	[7]
Tarattia gutama	NC_039833	15,266	81.2	[7]
Tacola larymna	NC_039887	15,503	80	[7]
Tacola eulimene	NC_039864	15,669	78.3	[7]
Limenitis amphyssa	NC_034235	15,164	80.9	Unpublished
Limenitis helmanni	NC_034754	15,178	81.1	Unpublished
Limenitis moltrechti	NC_034234	15,178	80.8	Unpublished
Limenitis sydyi	NC_034233	15,200	81.2	Unpublished
Limenitis populi	NC_039878	15,219	81	[7]
Limenitis ciocolatina	NC_039871	15,214	80	[7]
Limenitis arthemis	NC_039869	15,279	80.2	[7]
Limenitis archippus	NC_039868	15,220	80.9	[7]
Limenitis reducta	NC_039865	15,175	80.4	[7]
Limenitis cleophas	NC_039881	15,228	80.5	[7]
Limenitis camilla	NC_039866	15,129	80.7	[7]
Pandita sinope	NC_024398	15,257	81.4	[6]
Parasarpa dudu	NC_024405	15,236	81.1	[6]
Parasarpa albomaculata	NC_039875	15,272	81	[7]
Parasarpa zayla	NC_039698	15,269	80.4	[7]
Patsuia sinensium	NC_036333	15,192	81.2	[44]
Sumalia daraxa	KF590549	15,173	80.1	[6]
Sumalia zulema	NC_039859	15,297	80	[7]
Moduza procris	NC_036334	15,195	80.7	[44]

Species	GenBank no.	Size (bp)	Total A+T%	References
Adelpha iphiclus	NC_039696	15,263	80.6	-
Adelpha bredowii	NC_039885	15,187	80	[7]
Litinga cottini	NC_039877	15,205	80.3	[7]
Litinga mimica	NC_039874	15,183	80.7	[7]
Abrota ganga	NC_024404	15,356	81.2	[6]
Dophla evelina	NC_024400	15,320	81	[6]
Euthalia irrubescens	NC_024396	15,365	81	[6]
Lexias dirtea	NC_024399	15,250	81.3	[6]
Tanaecia julii	NC_024416	15,316	81.2	[6]
Auzakia danava	NC_039882	15,367	80.6	[7]
Chalinga elwesi	NC_039867	15,214	81.2	[7]
Seokia pratti	NC_039861	15,290	81.6	[7]
Bhagadatta austenia	NC_024413	15,615	79.2	[6]
Neptis philyra	NC_024419	15,164	80.2	[6]
Neptis soma	NC_024401	15,130	80	[6]
Pantoporia hordonia	NC_024402	15,603	80.7	[6]
Phaedyma columella	NC_036332	15,197	81.3	[44]
Parthenos sylvia	NC_024417	15,249	81.1	[6]
Athyma zeroca	OK393685	15,247	80.7	This study
Neptis thisbe	OK393687	15,188	79.1	This study
Neptis obscurior	OK393686	15,172	79.5	This study
Aldania raddei	OK393684	16,384	79.8	This study

Table S2. Mitogenomic organization of *Neptis thisbe*, *Neptis obscurior*, *Athyma zeroca* and *Aldania raddei*.

Gene	position		Size (bp)	Intergenic nucleotides	codon		Strand
	from	to			start	stop	
trnM	1/1/1/1	68/68/68/68	68/68/68/68				J
trnI	69/69/71/69	134/134/138/134	66/66/68/66	0/0/2/0			J
trnQ	132/132/136/132	200/200/204/200	69/69/69/69	-3/-3/-3/-3			N
nad2	257/258/254/256	1261/1262/1264/1263	1005/1005/1011/1008	56/57/49/55	ATT/ATT/ATT/ATT	TAA/TAA/TAA/TAA	J
trnW	1260/1261/1263/1262	1326/1327/1330/1328	67/67/68/67	-2/-2/-2/-2			J
trnC	1319/1320/1323/1321	1381/1382/1385/1385	63/63/63/65	-8/-8/-8/-8			N
trnY	1384/1385/1390/1388	1448/1449/1455/1452	65/65/66/65	2/2/4/2			N
cox1	1452/1453/1463/1456	2982/2983/2993/2986	1531/1531/1531/1531	3/3/7/3	CGA/CGA/CGA/CGA	T/T/T/T	J
trnL2	2983/2984/2994/2987	3049/3050/3060/3053	67/67/67/67	0/0/0/0			J
cox2	3050/3051/3062/3054	3725/3726/3740/3729	676/676/679/676	0/0/1/0	ATT/ATT/ATG/ATT	T/T/T/T	J
trnK	3726/3727/3741/3730	3796/3797/3811/3800	71/71/71/71	0/0/0/0			J
trnD	3796/3797/3811/3800	3861/3862/3876/3865	66/66/66/66	-1/-1/-1/-1			J
atp8	3862/3863/3877/3866	4032/4033/4041/4033	171/171/165/168	0/0/0/0	ATA/ATA/ATT/ATA	TAA/TAA/TAA/TAA	J
atp6	4026/4027/4035/4027	4703/4704/4712/4704	678/678/678/678	-7/-7/-7/-7	ATG/ATG/ATG/ATG	TAA/TAA/TAA/TAA	J
cox3	4703/4708/4718/4712	5491/5496/5506/5500	789/789/789/789	-1/3/5/7	ATG/ATG/ATG/ATG	TAA/TAA/TAA/TAA	J
trnG	5494/5499/5509/5503	5560/5565/5575/5569	67/67/67/67	2/2/2/2			J
nad3	5561/5566/5576/5570	5914/5919/5929/5923	354/354/354/354	0/0/0/0	ATT/ATT/ATT/ATT	TAG/TAG/TAG/TAG	J
trnA	5913/5919/5928/5922	5979/5983/5993/5987	67/66/66/66	-2/-2/-2/-2			J
trnR	6005/5995/5993/6010	6067/6057/6055/6075	63/63/63/66	25/11/-1/22			J
trnN	6069/6858/6056/6076	6134/6124/6120/6140	66/67/65/65	1/0/0/0			J
trnS1	6133/6123/6119/6139	6195/6183/6179/6200	63/61/61/62	-2/-2/-2/-2			J
trnE	6197/6185/6182/6202	6265/6252/6248/6269	69/68/67/68	1/1/2/1			J
trnF	6264/6251/6247/6268	6332/6320/6311/6334	69/70/65/67	-2/-2/-2/-2			N
nad5	6333/6321/6312/6335	8064/8058/8043/8066	1732/1738/1732/1732	0/0/0/0	ATA/ATA/ATT/ATA	T/T/T/T	N
trnH	8065/8062/8044/8067	8135/8132/8111/8137	71/71/68/71	0/3/0/0			N

Gene	position		Size (bp)	Intergenic nucleotides	codon		Strand
	from	to			start	stop	
nad4	8136/8130/8112/8138	9474/9468/9450/9476	1339/1339/1339/1339	0/-3/0/0	ATG/ATG/ATG/ATG	T/T/T/T	N
nad4L	9474/9468/9450/9476	9758/9752/9734/9760	285/285/285/285	-1/-1/-1/-1	ATG/ATG/ATG/ATG	TAA/TAA/TAA/TAA	N
trnT	9769/9766/9744/9775	9833/9830/9808/9839	65/65/65/65	10/13/9/14			J
trnP	9834/9831/9809/9840	9898/9895/9872/9904	65/65/64/65	0/0/0/0			N
nad6	9910/9904/9875/9916	10428/10422/10405/10434	519/519/531/519	11/8/2/11	ATA/ATA/ATT/ATA	TAA/TAA/TAA/TAA	J
cytb	10428/10422/10405/10434	11579/11573/11556/11585	1152/1152/1152/1152	-1/-1/-1/-1	ATG/ATG/ATG/ATG	TAG/TAA/TAA/TAA	J
trnS2	11587/11584/11560/11598	11652/11649/11625/11665	66/66/66/68	7/10/3/12			J
nad1	11666/11660/11624/11664	12607/12601/12580/12620	942/942/957/957	13/10/-2/-2	ATG/ATG/ATG/ATG	TAA/TAA/TAA/TAA	N
trnL1	12609/12606/12582/12622	12676/12673/12649/12689	68/68/68/68	1/4/1/1			N
rrnL	12677/12674/12650/12690	13990/13989/13978/14004	1314/1316/1329/1315	0/0/0/0			N
trnV	13991/13990/13979/14005	14058/14057/14043/14072	68/68/65/68	0/0/0/0			N
rrnS	14059/14055/14044/14073	14781/14778/14818/14847	723/724/775/775	0/-3/0/0			N
CR	14782/14779/14819/14848	15188/15172/15247/16348	407/394/429/1501	0/0/0/0			

Table S3. Nucleotide compositions in regions of the *Neptis thisbe*, *Neptis obscurior*, *Athyma zeroca* and *Aldania raddei* mitochondrial genomes.

Species	Whole genome		AT-skew	GC-skew	PCGs		tRNAs		rRNAs		CR	
	Size (bp)	AT%			Size(bp)	AT%	Size(bp)	AT%	Size(bp)	AT%	Size(bp)	AT%
N. thisbe	15188	79.1	-0.039	-0.2115	11173	77.5	1469	80	2037	84.1	407	94.1
N. obscurior	15172	79.5	-0.036	-0.2195	11179	77.9	1467	80.6	2040	84.1	394	95.2
A. zeroca	15247	80.7	-0.048	-0.1917	11203	79.3	1456	81.2	2104	84.9	429	93
A. raddei	16348	79.8	-0.033	-0.1921	11188	78.5	1470	81.7	2090	84.5	1501	80

Table S4. Best partitioning scheme and nucleotide substitution models for different datasets selected by PartitionFinder.

Dataset	Partition	Subset Partitions	Model
PCG123-BI	P1	*atp6*_pos1, *nad3*_pos1, *cox3*_pos1, *cytb*_pos1	GTR+I+G
	P2	*cox1*_ pos2, *cox3*_ pos2, *cox2*_ pos2, *cytb*_ pos2, *atp6*_ pos2	GTR+I+G
	P3	*cox1*_ pos3, *cox3*_ pos3, *cox2*_ pos3, *atp6*_ pos3	GTR+G
	P4	*nad6*_ pos2, *atp8*_ pos2, *atp8*_ pos1	HKY+I+G
	P5	*nad2*_ pos3, *atp8*_ pos3, *nad6*_ pos3	GTR+G
	P6	*cox2*_ pos1, *cox1*_ pos1	GTR+I+G
	P7	*nad3*_ pos3, *cytb*_ pos3	HKY+G
	P8	*nad5*_ pos1, *nad1*_ pos1	GTR+I+G
	P9	*nad4L*_ pos2, *nad1*_ pos2, *nad4*_ pos2, *nad5*_ pos2	GTR+I+G
	P10	*nad5*_ pos3, *nad1*_ pos3	GTR+G
	P11	*nad6*_ pos1, *nad2*_ pos1, *nad4*_ pos1, *nad4L*_ pos1	GTR+I+G
	P12	*nad2*_ pos2, *nad3*_ pos2	HKY+I+G
	P13	*nad4L*_ pos3, *nad4*_ pos3	GTR+G
PCG123-ML	P1	*nad4L*_ pos1, *atp6*_ pos1, *cytb*_ pos1, *cox3*_ pos1, *cox2*_ pos1, *cox1*_ pos1	GTR+I+G
	P2	*cox1*_ pos2, *nad2*_ pos2, *nad3*_ pos2, *cox3*_ pos2, *cox2*_ pos2, *cytb*_ pos2, *atp6*_pos2	GTR+I+G
	P3	*cox*1_ pos3, *cox2*_ pos3, *atp6*_ pos3	TVM+G
	P4	*nad6*_ pos2, *atp8*_ pos2, *nad3*_ pos1, *atp8*_ pos1, *nad6*_ pos1, *nad2*_ pos1	GTR+I+G
	P5	*nad2*_ pos3, *atp8*_ pos3	TIM+G
	P6	*nad6*_ pos3, *nad3*_ pos3, *cox3*_ pos3, *cytb*_ pos3	TIM+I+G
	P7	*nad4*_ pos1, *nad5*_ pos1, *nad1*_ pos1	TVM+I+G
	P8	*nad4L*_ pos2, *nad1*_ pos2, *nad5*_ pos2, *nad4*_ pos2	GTR+I+G
	P9	*nad1*_ pos3	TIM+I+G
	P10	*nad4L*_ pos3, *nad4*_ pos3	GTR+G
	P11	*nad5*_ pos3	TIM+I+G
PCG123R-BI	P1	*nad3*_ pos1, *atp6*_ pos1, *cox3*_ pos1, *cytb*_ pos1	GTR+I+G
	P2	*cox1*_ pos2, *cox3*_ pos2, *cox2*_ pos2, *cytb*_ pos2, *atp6*_ pos2	GTR+I+G
	P3	*cox1*_ pos3, *cox2*_ pos3, *atp6*_ pos3	GTR+G

Dataset	Partition	Subset Partitions	Model
	P4	*nad6*_ pos2, *atp8*_ pos2, *atp8*_ pos1	HKY+I+G
	P5	*nad2*_ pos3, *atp8*_ pos3, *nad6*_ pos3	GTR+G
	P6	*cox2*_ pos1, *cox1*_ pos1	GTR+I+G
	P7	*nad3*_ pos3, *cox3*_ pos3, *cytb*_ pos3	HKY+I+G
	P8	*nad5*_ pos1, *nad1*_ pos1	GTR+I+G
	P9	*nad4L*_ pos2, *nad1*_ pos2, *nad4*_ pos2, *nad5*_ pos2	GTR+I+G
	P10	*nad1*_ pos3, *nad5*_ pos3	GTR+G
	P11	*nad6*_ pos1, *nad2*_ pos1, *nad4*_ pos1, *nad4L*_ pos1	GTR+I+G
	P12	*nad2*_ pos2, *nad3*_ pos2	HKY+I+G
	P13	*nad4L*_ pos3, *nad4*_ pos3	GTR+G
	P14	*rrnL*, *rrnS*	GTR+I+G
PCG123R-ML	P1	*nad4L*_ pos1, *atp6*_ pos1, *cytb*_ pos1, *cox3*_ pos1, *cox2*_ pos1, *cox1*_ pos1	GTR+I+G
	P2	*cox1*_ pos2, *nad2*_ pos2, *nad3*_ pos2, *cox3*_ pos2, *cox2*_ pos2, *cytb*_ pos2, *atp6*_pos2	GTR+I+G
	P3	*nad3*_ pos3, *cox1*_ pos3, *cox2*_ pos3, *atp6*_ pos3	TVM+G
	P4	*nad6*_ pos2, *atp8*_ pos2, *nad3*_ pos1, *atp8*_ pos1, *nad6*_ pos1, *nad2*_ pos1	GTR+I+G
	P5	*nad6*_ pos3, *nad2*_ pos3, *atp8*_ pos3	TIM+G
	P6	*cox3*_ pos3, *cytb*_ pos3	TIM+I+G
	P7	*nad4*_ pos1, *nad5*_ pos1, *nad1*_ pos1	TVM+I+G
	P8	*nad4L*_ pos2, *nad1*_ pos2, *nad5*_ pos2, *nad4*_ pos2	GTR+I+G
	P9	*nad1*_ pos3	TIM+I+G
	P10	*nad4L*_ pos3, *nad4*_ pos3	GTR+G
	P11	*nad5*_ pos3	TIM+G
	P12	*rrnL*, *rrnS*	GTR+I+G
PCG123RT-BI	P1	*trnM*, *trnK*, *nad3*_ pos1, *atp6*_ pos1, *cytb*_ pos1, *cox3*_ pos1	GTR+I+G
	P2	*cox1*_ pos2, *cox3*_ pos2, *cox2*_ pos2, *cytb*_ pos2, *atp6*_ pos2	GTR+I+G
	P3	*cox1*_ pos3, *cox2*_ pos3, *atp6*_ pos3	GTR+G
	P4	*nad2*_ pos1, *nad6*_ pos1, *atp8*_ pos1	GTR+I+G
	P5	*nad6*_ pos2, *atp8*_ pos2, *trnS1*	GTR+I+G
	P6	*nad2*_ pos3, *atp8*_ pos3, *nad6*_ pos3	GTR+G

Dataset	Partition	Subset Partitions	Model
	P7	*cox2*_ pos1, *cox1*_ pos1, *trnL2*	GTR+I+G
	P8	*nad3*_ pos3, *cox3*_ pos3, *cytb*_ pos3	HKY+I+G
	P9	*nad5*_ pos1, *nad1*_ pos1, *trnL1*, *trnT*, *trnF*, *trnA*, *trnC*, *nad4L*_ pos1, *nad4*_ pos1	GTR+I+G
	P10	*nad4L*_ pos2, *nad1*_ pos2, *nad5*_ pos2, *nad4*_ pos2	GTR+I+G
	P11	*nad5*_ pos3, *nad1*_ pos3	GTR+I+G
	P12	*nad3*_ pos2, *nad2*_ pos2	HKY+I+G
	P13	*nad4L*_ pos3, *nad4*_ pos3	GTR+G
	P14	*trnN*, *trnS2*, *trnE*, *trnG*, *trnW*, *trnY*, *rrnL*, *rrnS*	GTR+I+G
	P15	*trnH*, *trnQ*, *trnP*, *trnD*, *trnI*, *trnR*, *trnV*	HKY+I+G
PCG123RT-ML	P1	*cox2*_ pos1, *cox1*_ pos1, *trnL2*, *atp6*_ pos1, *cytb*_ pos1, *cox3*_ pos1, *trnM*, *trnK*	GTR+I+G
	P2	*cox1*_ pos2, *nad2*_ pos2, *nad3*_ pos2, *cox3*_ pos2, *cox2*_ pos2, *atp6*_ pos2, *cytb*_pos2	GTR+I+G
	P3	*cox1*_ pos3, *cox2*_ pos3, *atp6*_ pos3	TVM+G
	P4	*nad6*_ pos2, *atp8*_ pos2, *trnS1*, *atp8*_ pos1, *trnT*, *nad3*_ pos1	TRN+I+G
	P5	*nad6*_ pos3, *nad2*_ pos3, *atp8*_ pos3	TIM+G
	P6	*nad3*_ pos3, *cox3*_ pos3, *cytb*_ pos3	K81UF+I+G
	P7	*trnQ*, *trnH*, *nad1*_ pos1, *nad5*_ pos1, *trnL1*, *nad4*_ pos1, *trnF*, *nad4L*_ pos1	TVM+I+G
	P8	*nad4L*_ pos2, *nad1*_ pos2, *nad5*_ pos2, *nad4*_ pos2	GTR+I+G
	P9	*nad1*_ pos3	TIM+I+G
	P10	*nad6*_ pos1, *nad2*_ pos1	GTR+I+G
	P11	*nad4L*_ pos3, *nad4*_ pos3	GTR+G
	P12	*nad5*_ pos3	TIM+G
	P13	*trnY*, *rrnL*, *rrnS*, *trnE*, *trnG*, *trnA*, *trnC*	GTR+I+G
	P14	*trnI*, *trnW*, *trnD*, *trnN*, *trnS2*, *trnP*, *trnV*, *trnR*	K81UF+I+G
